# *QuickStats:* Age-Adjusted Pedestrian* Death Rates,^†^ by Race/Ethnicity — National Vital Statistics System, United States, 2009 and 2018

**DOI:** 10.15585/mmwr.mm6939a7

**Published:** 2020-10-02

**Figure:**
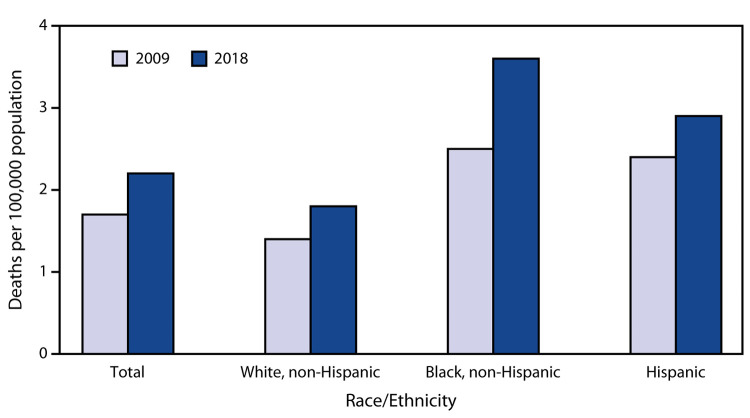
The age-adjusted pedestrian death rate increased from 1.7 per 100,000 in 2009 to 2.2 in 2018. This increase was seen in each racial/ethnic group: from 1.4 to 1.8 per 100,000 for non-Hispanic White persons, from 2.5 to 3.6 for non-Hispanic Black persons, and from 2.4 to 2.9 for persons of Hispanic origin. In both 2009 and 2018, non-Hispanic White persons had the lowest death rate; in 2018, the rate was highest for non-Hispanic Black persons.

**Sources:** National Center for Health Statistics, National Vital Statistics System, mortality data, 2009 and 2018; CDC WONDER online database. https://wonder.cdc.gov/ucd-icd10.html.

